# Community promotion and application of Wuqinxi combined with brief behavioral therapy for insomnia: A study protocol

**DOI:** 10.1097/MD.0000000000028046

**Published:** 2021-12-10

**Authors:** Yongliang Zheng, Yiyu Qin, Yumeng Lyu, Liangliang Li, Ya Chen, Zhaojuan Yao

**Affiliations:** a Jiangsu Vocational College of Medicine, Yancheng, Jiangsu, China; b Community Health Service Center of Dongting Lake Street, China; c Community Health Service Center of Xindu Street, Yancheng, Jiangsu, China.

**Keywords:** behavioral therapy, insomnia, traditional Chinese medicine

## Abstract

**Background:**

Brief behavioral therapy for insomnia (BBT-I) has been proven to be a simple and effective alternative to cognitive behavioral therapy. However, low adherence limits the application in Chinese primary medical institutions, resulting in delayed or irregular treatment for many patients. This study aimed to explore the efficacy of traditional Chinese medicine external treatments on the adherence to behavioral therapy for insomnia in Chinese primary healthcare institutions, with a particular focus on patients who live in regions with weak healthcare systems.

**Methods:**

This randomized controlled clinical trial will be conducted in primary medical institutions and will recruit 98 adult participants with insomnia. BBT-I will be used as the base treatment. The participants will be divided into experimental (combined with Wuqinxi and other traditional Chinese medicine [TCM] external treatment n = 49) and control (combined with trazodone treatment, n = 49) groups, and each group will be treated for 4 consecutive weeks. The severity index of insomnia will be used as the main indicator of disease evaluation, with an 8-point reduction in the score considered as effective and a score <8 considered as cured. The secondary indicators of the disease evaluation will include the Pittsburgh sleep quality index, Zung's self-rating anxiety scale, Zung's self-rating depression scale, treatment adherence, and adverse event reports. All participants will be followed up at the time of enrollment, 4 weeks after treatment, and 3 months after the end of treatment.

**Discussion:**

This clinical trial will provide evidence for the efficacy of traditional Chinese medicine external treatment on the adherence to behavioral therapy for insomnia in primary medical institutions. This cheap and accessible model may benefit insomnia patients in medically underserved areas.

**Trial registration:**

Chinese Clinical Trial Registry ChiCTR2100042845. Registered on 30 January 2021, dataset: http://www.chictr.org.cn/showproj.aspx?proj=65691. Official scientific title of the research topic: Wuqinxi and other external treatment of Chinese Medicine combined with brief behavior therapy for insomnia.

## 1 Introduction

### 1.1 Background

According to relevant epidemiological studies, approximately 35% to 50% of adults have experienced insomnia,^[1]^ and 12% to 20% of adults meet the diagnostic criteria for insomnia.^[2,3]^ Persistent insomnia increases the risk of physical or mental illness, traffic accidents, and can induce drug or alcohol abuse.^[4–6]^ Worldwide, cognitive behavior therapy for insomnia (CBT-I) has been accepted as the first-line treatment for insomnia.^[7–10]^ However, previous studies have reported that insomnia is mainly treated by medication or sleep hygiene education in primary healthcare institutions.^[11,12]^ To change the status quo, some researchers have achieved success with the development of the brief behavioral therapy for insomnia (BBT-I) model^[13,14]^ or other CBT-I variants,^[15–18]^ which are simplified CBT-I with behavioral therapy as the core model.^[13–20]^ Although the development of these models has allowed CBT-I to be conducted in primary healthcare institutions, low adherence to behavior therapy is an important problem that has limited its promotion.^[21]^ Current research has considered that the main reasons for the low adherence are low efficacy caused by daytime fatigue,^[22,23]^ anxiety, and depression.^[24]^ During treatment, the steps with the lowest adherence were found to be restricting daytime naps, followed by fixed wake-up time, limiting time in bed, and getting out of bed if not falling asleep.^[18]^

In the current research, traditional Chinese Medicine external treatment (TCMET) refers to *Wuqinxi* (also known as five-animal qigong exercise), Chinese medicine massage (*Tuina*), and herbal foot baths, all of which have a long history of use in the treatment of insomnia or depression and will be scheduled in BBT-I to enhance the adherence of the participants. Modern relevant clinical studies have shown the therapeutic effect of *Wuqinxi* on sleep and mental illness.^[25–30]^ Additionally, Chinese medicine massage and herbal foot bath have been reported to improve fatigue,^[31–34]^ and these external therapies have a strong cultural identity in China. Theoretically, primary healthcare institutions in China should be able to perform BBT-I and the above mentioned external traditional Chinese medicine (TCM) treatments. We hypothesize that a combination of BBTI and TCMET improves the adherence and efficacy of behavioral therapy for patients with insomnia in community medical institutions. Additionally, our early exploration found that the loss of follow-up rate was very high in community patients treated only with behavior therapy, and the combination of drugs can effectively ensure the adherence of patients to follow-up. Some clinical and review studies have reported that the antidepressant, trazodone, may have a synergistic effect on CBT-I.^[35–38]^ As Trazodone is legally prescribed by physicians in primary medical institutions in the Chinese community, this trial uses trazodone as a comparator.

### 1.2 Objectives

The aim of this study is to explore whether the combination of TCMET and BBT-I is safer and more effective than the combination of trazodone and BBT-I in improving adherence to behavioral therapy.

### 1.3 Trial design

This clinical trial will adopt an exploratory two-group parallel and randomized control design using either TCMET combined with BBT-I (experimental group) or oral trazodone treatment combined with BBT-I (control group). Participants (n = 98) will be randomly divided into the experimental (n = 49) and control (n = 49) groups at a ratio of 1:1. The trial and protocol adhere to SPIRIT recommendations.^[39]^

## 2 Methods

### 2.1 Study setting

Participants will be recruited and treated at the Community Health Service Center, Xindu Sub-district, Yancheng City (Jiangsu Province, China), the Community Health Service Center of Dongting Lake Street (Jiangsu Province, China). The measurement and follow-up of the trial will be performed in Jiangsu Vocational College of Medicine in Yancheng City, Jiangsu Province, China, which specializes in training medical staff for primary medical institutions.

### 2.2 Eligibility criteria

Patients with chronic insomnia will be recruited from the primary healthcare centers where the general practitioners will perform the interventions. Individuals with co-morbidities will only be excluded if there is a life-threatening situation, or the use of study drugs is prohibited. Moreover, individuals who use psychotropic drugs (e.g., anti-anxiety drugs, antidepressants, benzodiazepines, or non-benzodiazepine sleep aids) will not be automatically excluded. Patients who have taken a stable dose of selective serotonin reuptake inhibitor or serotonin and norepinephrine reuptake inhibitor drugs (for at least 3 months), have at least partial relief of mood or anxiety disorders, and who meet the selection criteria will be included in the study. Patients who are taking monoamine oxidase inhibitors, tricyclic antidepressants, or atypical antidepressants will be excluded, even if they are in remission, due to the possibility that these drugs will confuse the interpretation of the study results.^[40]^

#### 2.2.1 Inclusion criteria

The inclusion criteria are as follows:

1. Patients who meet the diagnostic criteria in the *Diagnostic and Statistical Manual of Mental Disorders, Fifth Edition*^[41]^ and in the *Criteria for Disease Diagnosis in TCM and TCM Therapeutic Efficacy*.^[42]^
2. Patients who are males or females >18 years old.
3. Patients who have difficulties in falling asleep or maintaining sleep for ≥1 month despite having enough opportunities to sleep, with an incubation period of sleep onset or the waking time after sleep onset exceeding 30 min (occurring at least 3 nights a week).
4. Patients with >10 insomnia severity index (ISI) total scores, indicating at least mild insomnia.
5. Patients who participate in this study voluntarily, can cooperate in completing various scale evaluations during the treatment period and in treatment, and provide informed consent.

#### 2.2.2 Exclusion criteria

The exclusion criteria are as follows:

1. *Patients with or suspected of having severe mental illness or suicidal tendencies.*
2. *Patients with substance use disorders (including addition or abuse of substances such as alcohol, caffeine, sedative, and psychoactive drugs).*
3. *Patients with history of epilepsy, mania, bipolar disorder, or parasomnia.*
4. *Patients currently using drugs that may cause insomnia (e.g., steroids) and suspected of having severe sleep apnea (≥5 positive items in the snoring, tiredness, observed apnea, high blood pressure, body mass index, age, neck circumference, and male sex [STOP-Bang] questionnaire),* ^[43]^ *restless legs syndrome, circadian rhythm sleep disorders, personal or family (first degree relatives) parasomnia history.*
5. *Patients who suffer from severe diseases, other exercise contraindications, or special conditions that are considered unsuitable for Wuqinxi* **qigong exercise; who are not suitable for sleep restriction and stimulation control therapy; or who have contraindications to trazodone and liver and kidney dysfunction.**
6. **Patients who have practiced** *Wuqinxi* **for a long time, have received or are receiving CBT-I treatment (including BT-I, online CBT-I, and self-help CBT-I) in other medical institutions.**

### 2.3 Evaluation standards

#### 2.3.1 Structured clinical interview

A structured clinical interview to screen potential participants has been developed by our group. The interview content is in accordance with the diagnostic and statistical manual of mental disorders, fifth edition^[41]^ and the Duke structured interview for sleep disorder^[44]^ and gives full consideration to the actual situation of primary medical institutions in the Chinese community. In the absence of a gold standard interview tool,^[41,45]^ we have developed a questionnaire to obtain the history of sleep behavior and history of potential sleep disease of the candidates to allow physicians to rapidly diagnose insomnia and screen for other comorbid sleep disorders (i.e., sleep deprivation, lethargy, circadian rhythm sleep-wake disorder, severe sleep apnea, restless legs syndrome) to in primary medical institutions in China, and to facilitate the evaluation of eligibility for inclusion in this study.

#### 2.3.2 Sleep diary

The sleep diary based on the web-based follow-up system will be used to obtain subjective estimates of sleep and wake-up time of the participants daily. Each participant will be asked to cooperate with the follow-up staff to report the information about their sleep the night before, including bedtime, sleep onset latency, number and duration of night-time awakenings, last awakening time, wake-up time, subjective evaluation of sleep quality, and use of sleep aid drugs. A 1-week sleep diary will be obtained in the first week after the study enrollment. During the telephone follow-up, the follow-up staff will give necessary guidance and supervision to ensure the patient's adherence to behavioral therapy.

#### 2.3.3 BBT-I adherence questionnaire

Good adherence to therapy is key to the curative effect of behavioral therapy.^[46]^ One of the main hypotheses of this study is that both the external TCM and trazodone can help patients to improve their therapeutic adherence. Unfortunately, there are currently few relevant studies and no gold standard for evaluating adherence with CBT-I or its brief model.^[22,47,48]^ To evaluate the adherence of participants, our team devised the BBT-I adherence questionnaire based on the contents of sleep restriction and stimulus control, which focuses on the patient's behavioral therapy adherence in the past week, including insistence on not going to bed at night without sleeping, getting up early in the morning, not going to bed during the day, not staying in bed at night after attempting to sleep for 30 minutes, and not doing anything unrelated to sleep or having sex (especially using cell phone and thinking). Each content will be scored from 0 to 3 as follows: score 0, difficult to accomplish; score 1, accomplish less than half the time; score 2, accomplish more than half the time; score 3, accomplish most of the time.

### 2.4 Outcomes

#### 2.4.1 Main outcome indicator

The ISI will be used to evaluate the improvement of insomnia at the baseline level before the treatment, at the end of the 4-week treatment period, and 3 months after the end of treatment. A reduction in the ISI score of ≥8 points is considered effective, and an ISI score ≤8 is considered cured.^[49,50]^ The BBT-I adherence questionnaire will be used to evaluate the efficacy of TCMET or trazodone on the adherence of BBT-I.

#### 2.4.2 Secondary outcome indicators

The secondary indicators of the disease evaluation will include the pittsburgh sleep quality index,^[51]^ Zung's self-rating anxiety scale, Zung's self-rating depression scale,^[52]^ patient's sleep quality, mental state, and possible adverse reactions of the participants recorded during the follow-up.

### 2.5 Recruitment and consent

Through community clinics, Dr. Yongliang Zheng's online workstation, the Good Doctor online app, promotion of community physical examinations, community health lectures, and other forms, patients with insomnia will be introduced and recruited to the clinical trial. General practitioners in primary healthcare centers use structured clinical interviews to identify potential participants meeting eligibility criteria. General practitioners then inform a monitoring group doctor to obtain informed consent from potential participants, and complete further screening assessments. The research group will obtain written informed consent from all participants during the enrollment, and the participants recruited on the online platform will be required to print and sign the electronic version of the informed consent form and send it back to the research group for archiving.

### 2.6 Randomization

Eligible participants will be randomized into the experimental group or control group using a complete randomization scheme generated in advance. Specifically, the randomization scheme will be generated in the computer program Stata/SE15.1 by a member of the research staff (Liangliang Li) who is not involved in recruitment or intervention and is not the principal investigator. The assignment of participants will be hidden using sequentially numbered-sealed e-mails, which will only be opened by the researchers after the informed consent form is signed. The study grouping will be revealed at the same time to both the patient and researchers.

### 2.7 Interventions

The standard treatment for insomnia will be BBT-I,^[14]^ including sleep restriction, stimulation control, and sleep hygiene education.^[9,53–55]^ The implementation of sleep restriction and stimulation control will be guided by trained community physicians in the outpatient clinics. The corresponding bedtime, wake-up time, and other related recommendations for behavioral treatment will be given to the participants according to their sleep conditions and will be adjusted during the follow-up based on the 1-week sleep diary. Sleep hygiene education will be conducted in the form of health lectures and online free consultations. Related video resources will also be distributed to allow participants to partake in further study to better understand their condition. Although complete CBT-I may be the best treatment option, BBT-I is a standard treatment that can be conducted in primary medical institutions in the Chinese community because of its simple and easy implementation.

Considering the various non-adherence issues that occur in the implementation of BBT-I, we will schedule TCMET in BBT-I to improve patient's behavioral adherence in the experimental group. TCMET include *Wuqinxi* qigong exercise, Chinese medicine massage, and herbal foot bath.

1. *Wuqinxi* qigong will be exercised when participants who fail to fall asleep half an hour after going to bed, and cannot fall asleep again half an hour after waking up early. *Wuqinxi* consists of 5 parts: Tiger Exercise, Ape Exercise, Bear Exercise, Crane Exercise, and Deer Exercise. Exercise recommendation is based on the 5 elements theory of traditional Chinese medicine. According to the 5 elements theory, Tiger Exercise corresponds to anger, so practicing Tiger Exercise can ease the anger mood. Similarly, Ape Exercise corresponds to joy, Bear Exercise corresponds to thinking, Crane Exercise corresponds to sadness, and Deer Exercise corresponds to panic. Therefore, for participants who are unable to fall asleep due to irritability or anger, he or she will be suggested to get out of bed to practice Tiger Exercise. For participants who are unable to fall asleep due to joy, he or she will be suggested to get out of bed to practice Ape Exercise. For participants who are unable to fall asleep due to worries, they will be recommended to get up to practice Bear Exercise. For participants who are unable to fall asleep due to sadness, he or she will be required to get out of bed to practice Crane Exercise. For participants who are unable to fall asleep due to panic, he or she will be required to get out of bed to practice Deer Exercise.^[56]^
2. Participants who have difficulty cooperating with sleep restriction at night (postponing bedtime) will be prescribed Chinese medicine foot bath by an attending physician dialectically based on the TCM theory, with no uniform limitations. The time of the Chinese medicine foot bath will be half an hour before bedtime at night as recommended by the physicians.
3. Participants who feel tired during the day and have difficulty cooperating with the implementation of daytime sleep restriction will be given Chinese massage, with no specific restrictions on the duration, acupoints, and techniques of the massage. The therapist will implement the Chinese massage according to the specific conditions of the participants.

Participants in the control group will be given standard treatment based on BBT-I combined with oral trazodone medication (25–150 mg trazodone as an adjuvant therapy, 30 minutes before going to bed). Trazodone is selected because it is one of the most common drug prescriptions for insomnia in clinical practice.^[57–60]^ If the patient refuses to take trazodone, we will provide BBT-I only and record it.

### 2.8 Implementation procedures

Once the research candidates have completed several written screening measures, those who meet the inclusion criteria will be screened and evaluated, with the results used as the baseline assessment data. The candidates will be randomly treated in the experimental (i.e., BBT-I combined with TCMET) and control (i.e., BBT-I combined with oral trazodone medication) groups, all of whom must complete the 4 weeks of corresponding treatments. In the first week, the follow-up staff will assist the patient to complete a 1-week sleep diary by telephone, during which, the follow-up staff will provide the necessary guidance and supervision to ensure the participants’ behavioral therapy adherence. After the first week, the participants will be instructed to adjust the behavioral treatment plan. In the second and third weeks, the participants’ behavioral therapy adherence will be followed up with the BBT-I adherence questionnaire, and guidance to ensure behavioral therapy adherence will be given during the follow-up. At the end of the fourth week, sleep and mood-related scales will be evaluated, and the BBI-I adherence questionnaire will be performed again. Participants who demonstrate an improvement will continue maintenance therapy, and participants with no improvement over the 4-week study period will have their treatment plan adjusted or will be recommended to be referred to a higher-level hospital. During the treatment period, the treatment will be discontinued in participants who cannot cooperate with the treatment due to disease or other reasons.

**Figure 1:**
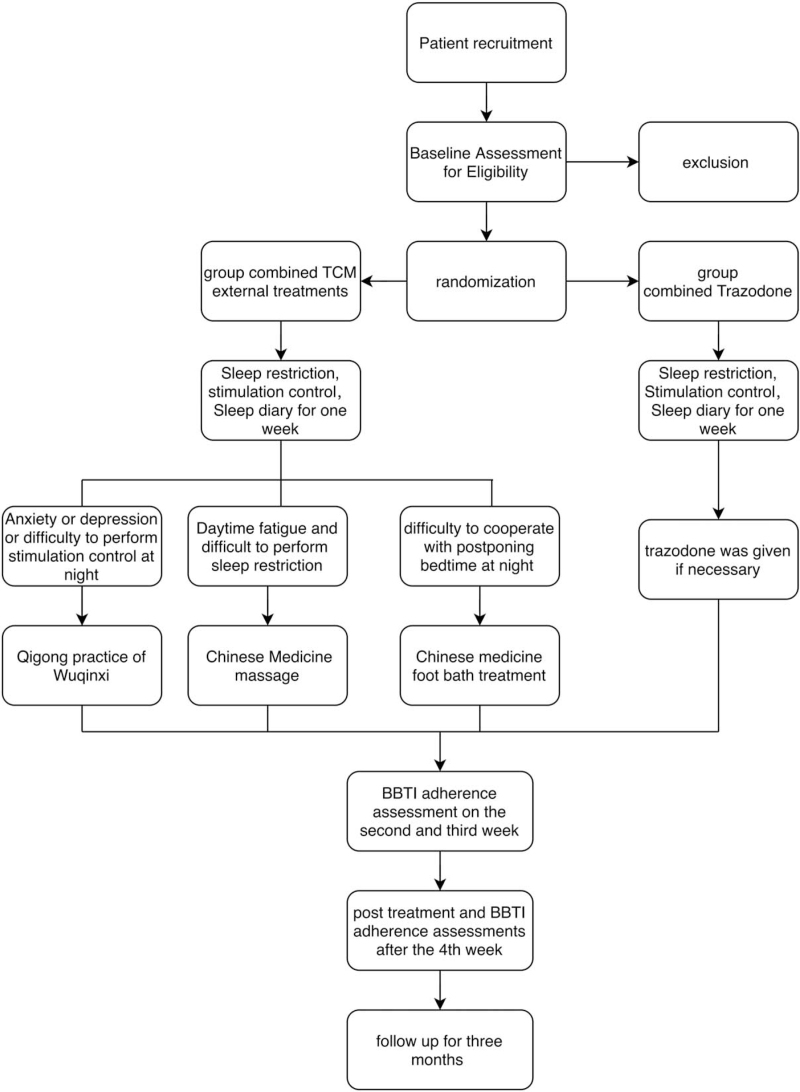
Overview of the study.

Three months after the end of the 4-week treatment, a telephone follow-up will be performed to evaluate the therapeutic efficacy. All follow-up tasks above will be conducted through the network chronic disease follow-up platform (NCDFP) developed by Jiangsu Vocational College of Medicine (Jiangsu Province, China), and will be completed by trained students through the telephone interview. Table 1 shows the participant timeline, and Figure 1 provides an overview of the study.

**Table 1:**
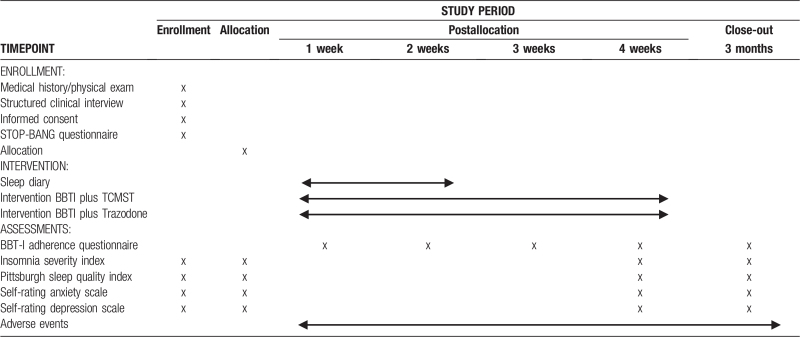
The schedule of enrollment, interventions and assessments.

### 2.9 Concomitant care

Participants who have been taking hypnotic drugs or antidepressants before enrollment will be permitted to continue taking them; however, the addition of hypnotic drugs or treatment other than the given interventions during trial will be prohibited if the participants did not take them before enrollment.

### 2.10 Data processing and analysis

#### 2.10.1 Data processing

The baseline demographics of the research subjects will be collected by the attending physician through paper-and-pen records at the time of recruitment and will be entered into the database. The data during the treatment and follow-up period will be collected by follow-up staff using the NCDFP, an online telephone survey, which will allow participants to perform this at their convenience. All participants will be reminded throughout the study to fill out the questionnaires during study visits. The follow-up process will be recorded in real time to verify the accuracy of the data. The project manager will be responsible for double checking and exporting the original data from the follow-up system to the required format for data analysis. After the study is completed, the pattern of missing data will be investigated to determine whether the data are completely missing at random or missing non-randomly.^[61]^ Our preliminary analysis will use a robust statistical model, and a sensitivity analysis of nonrandom missing data will be performed if necessary.^[62]^

#### 2.10.2 Data analysis

Analysis of the validity and reliability of the BBT-I adherence questionnaire: the adherence reliability of the behavioral therapy will evaluate the internal consistency of the behavioral therapy adherence through the standard Cronbach's α coefficient and the total correlation coefficient of the item. Convergence validity analysis will be based on the participants’ 1-week daily sleep diary, which will be compared with the consistency of the questions answered in the BBT-I adherence questionnaire to observe whether the questionnaire truly reflects the accuracy of the patient's adherence with various behavioral treatments. For example, for the question “Can you get up on time in the morning in the last week?” if the patient's answer is “accomplish less than half the time” then the patient should have gotten up on time 1 to 3 days during the last week; if the patient's answer is “accomplish more than half the time,” the sleep dairy of this patient should indicate that he or she has gotten up on time for 4 to 6 days in the last week; if the patient's answer is “not able to accomplish,” the sleep dairy of this patient should indicate that he or she had 0 days getting up on time in the last week; if the patient's answer is “accomplish most of the time,” the sleep diary of this patient should indicate that he or she has gotten up on time for all 7 days in the last week. Waking up on time is defined as getting up no more than 15 minutes before or after the agreed time to get up. Failure to meet the above criteria will be considered inconsistency between questionnaire's answer and the sleep diary. Lastly, the consistency ratio will be determined, and the correlation coefficient will be calculated.

Analysis of therapeutic efficacy within and between groups, and adherence analysis between the groups: the measurement data will be presented as the mean ± standard deviation, and the statistical inference will be performed using the *t*-test and rank-sum test. The count data will be presented as frequency (composition ratio), and Chi-Squared analysis or nonparametric analysis will be performed for exact probability or statistical inference, respectively. Sensitivity analyses will be used to assess the impact of missing outcomes, which include worst case assumptions and/or multiple imputation methods. Subgroup analysis will be used to analyze the main outcome and adherence according to associated medication (Trazodone used or not) and education level of the research subjects. SPSS 21.0 software (IBM, Armonk, NY) will be used for statistical analysis, which will adopt two-sided tests. *P* ≤ .05 will be considered to indicate statistically significant differences in the test.

#### 2.10.3 Sample size calculation

The main observation index of this study is the ISI, and the sample size is calculated based on the two independent sample *t*-test. The sample size adjustment is based on the pool variance.^[63]^ According to our previous reports,^[25,50]^ the mean effect size of the control group is 8.4. A mean effect size difference (δ) of an increase in 2.5 for the experimental group relative to the control group will be considered effective. The overall sample standard deviation of ISI in the previous study was 4.1.^[49]^ Assuming that the variances of the 2 groups are similar, in a two-sided test with α at .05, sample size ratio of the 2 groups at 1:1, the test power (1 − β) at 80%, the sample size is calculated to be 88. We anticipate 10% loss to follow-up or drop-off rate. Thus, t total sample size is calculated as 96.8, which is approximately 98 people, including 49 people in each of the 2 groups.

## 3 Composition and responsibilities of the coordinating center

This study is a multicenter study that will be designed, performed, and coordinated in Jiangsu Vocational College of Medicine, Community Health Service Center of Dongting Lake Street and the Community Health Service Center of Xindu Street. The study team will meet weekly. The trial is supported by:

1. Monitoring group: supervises the trial, audits data, takes medical responsibility for the participants, provides BBT-I, and trains the follow-up group.
2. Physician group: identifies potential recruits, acquires informed consent, provides medical treatment according to the study protocol.
3. Follow-up group: conducts follow-up by telephone and records data in the NCDFP, feeds back questions to the Physician group, and safeguards data.
4. Supportive group: performs trial registration, safety reports, and coordinates with each team.

## 4 Discussion

In the design of this study, we fully considered the limitations of the professional ability of physicians in the Chinese community, the limitations of the examination methods in the primary medical institutions, the limitations of sleep-related drug administration by the community physicians, the higher acceptance of some TCM non-pharmaceutical therapies among the patients, and problems such as rejection of Western medicines (e.g., sleeping pills) among Chinese patients. This study will explore the integration of TCMET, drugs, and regular follow-up care on BBT-I to improve behavioral therapy adherence in participants with insomnia, thereby achieving curative effects. The results of this study will provide clinical evidence for the implementation of CBT-I in primary healthcare institutions in China.

The innovations of this study are:

1. The research design, which fully considers the various real-world constraints of primary medical institutions in China.
2. The research, which adopts active telephone follow-up design for each behavioral therapy step to achieve better behavioral therapy adherence through reminding and supervising the participants.
3. Comparative analysis of the follow-up of behavioral therapy adherence and sleep diary, which determines the reliability and validity of the BBT-I adherence questionnaire to explore the effectiveness of the questionnaire in evaluating participants’ behavioral therapy adherence.
4. Accurate integration between *Wuqinxi* qigong exercise, Chinese massage, herbal foot bath, and behavioral treatment.
5. The dosage of trazodone used in the control group at 25 to 150 mg/day instead of a fixed drug/fixed dosage design.

Due to the high prevalence, morbidity, and social cost of insomnia, this study may generate new information that can have a direct impact on the model of promoting CBT-I for insomnia, benefiting insomnia patients and the society, especially for patients who live in regions with weak healthcare systems.

## 5 Experimental status

This clinical study is currently in the active recruitment phase. As of April 17, 2021, patient recruitment has been initiated and 25 subjects have been enrolled. This is protocol version number 2, January 30, 2021.

## 6 Declarations

### 6.1 Ethics approval and safety monitoring

The research protocol has been approved by the Chinese Ethics Committee of Registering Clinical Trials (ethical review number: ChiECRCT20200452) and has been filed at the corresponding community health service center.

The trial will be monitored by the Steering Committee and the Data and Safety Monitoring Board (DSMB). The DSMB comprises 3 independent researchers, 1 network data security expert, 1 psychiatrist, and 2 members of the follow-up group. The DSMB will be responsible for monitoring participant safety and data quality, and will have access to those interim results and make the final decision to terminate the trial.

Adverse events will be recorded during follow-up. In the event that participants report severe symptoms of depression, anxiety, or previously undiagnosed severe psychiatric conditions to follow-up staff, the staff will inform their clinical team and will recommend the patient to a higher-level hospital to psychiatry or clinical psychology if necessary. Serious harm is expected to be impossible. However, if harm does occur, the medical liability insurance will cover care for participants.

As a funding limitation, there is no independent audit. The Science and Technology Department and Audit Office of Jiangsu Vocational College of Medicine are responsible for performing audit work once a year.

In the event that any substantial modifications to the protocol occur, both the Chinese Ethics Committee of Registering Clinical Trials and participants will be notified. If necessary, additional consent will be requested and registered. Non-substantial amendments will be recorded and filed.

### 6.2 Confidentiality

Unique study numbers will be used to label the participants for the research data. The only dataset with participant identifier information is the NCDFP, which is used to follow-up and contact participants. The login ID and password will only be provided to the research team during the study period. The storage server system of the NCDFP is installed in the Jiangsu Vocational College of Medicine and is not publicly available. After the completion of the study, the main researchers will submit the paper-based original files and data to the Science and Technology Department of the College for preservation, and the medical records will be kept in the corresponding hospitals. Patient identity details will not be reported in the publication.

## Acknowledgments

The authors thank all participants in this trial, the consumer representative Aiping Wu for her valuable advice regarding the telephone follow-up model, and the DSMB members who have supported this project, particularly, Junzhi Lei, for his help with the NCDFP building. We thank LetPub (www.letpub.com) for its linguistic assistance during the preparation of the manuscript.

## Author contributions

**Conceptualization:** Yongliang Zheng, Yumeng Lyu, Yiyu Qin, Ya Chen, Zhaojuan Yao.

**Funding acquisition:** Yongliang Zheng, Yiyu Qin.

**Investigation:** Ya Chen, Zhaojuan Yao, Liangliang Li, Yongliang Zheng.

Software: Yiyu Qin

**Writing – review & editing:** Yongliang Zheng, Yumeng Lyu, Liangliang Li.

**Conceptualization:** Yongliang Zheng, Yiyu Qin, Yumeng Lyu, Ya Chen, Zhaojuan Yao.

**Funding acquisition:** Yongliang Zheng, Yiyu Qin.

**Investigation:** Yongliang Zheng, Liangliang Li, Ya Chen, Zhaojuan Yao.

**Methodology:** Yongliang Zheng.

**Software:** Yiyu Qin.

**Writing – original draft:** Yongliang Zheng, Yumeng Lyu.

**Writing – review & editing:** Yongliang Zheng, Yumeng Lyu, Liangliang Li.
